# Tumor Associated Neutrophils. Their Role in Tumorigenesis, Metastasis, Prognosis and Therapy

**DOI:** 10.3389/fonc.2019.01146

**Published:** 2019-11-15

**Authors:** Maria Teresa Masucci, Michele Minopoli, Maria Vincenza Carriero

**Affiliations:** Tumor Progression Unit, Department of Experimental Oncology, Istituto Nazionale Tumori Fondazione “G. Pascale” IRCCS, Naples, Italy

**Keywords:** tumor microenvironment, tumor associated neutrophils, tumorigenesis, angiogenesis, neutrophil-to-lymphocyte ratio

## Abstract

Tumor Associated Neutrophils (TANs) are engaged into the tumor microenvironment by cytokines and chemokines, can be distinguished according to their activation and cytokine status and effects on tumor cell growing in N1 and N2 TANs. N1 TANs exert an antitumor activity, by direct or indirect cytotoxicity. N2 TANs stimulate immunosuppression, tumor growth, angiogenesis and metastasis by DNA instability, or by cytokines and chemokines release. In tumor patients, either a high number of TANs and Neutrophil-to-Lymphocyte Ratio (NLR) do correlate with poor prognosis, and, so far, TAN counts and NLR can be regarded as biomarkers. Owing to the pivotal role of TANs in stimulating tumor progression, therapeutic strategies to target TANs have been suggested, and two major approaches have been proposed: (a) targeting the CXCL-8/CXCR-1/CXCR-2 axis, thereby blocking TANs or (b) targeting substances produced by polymorpho-nuclear cells that promote tumor growth. Many studies have been accomplished either *in vitro* and in animal models, whereas clinical studies are restrained, presently, due to the risk of inducing immunosuppression. In this review, we deeply discuss the anti-tumorigenic or pro-tumorigenic activity of TANs. In particular, TANs relevance in tumor prognosis and *in vitro* therapeutic strategies are widely described. On-going clinical trials, aimed to inhibit neutrophil recruitment into the tumor are also accurately debated.

## Introduction

Neutrophils represent 50–70% of the myeloid derived white circulating cells in human blood, and are mainly involved in the human innate immunity against invading pathogens (1) Following cytokine stimulation, neutrophils acquire the potentiality to polarize to antitumor (N1) or pro-tumor (N2) phenotype (2–4). Immune profile of N1 TANs is characterized by high levels of an TNFα, CCL3, ICAM-1 and low levels of Arginase axis, whereas N2 neutrophils are characterized by upregulation of the chemokines “CCL2, CCL3, CCL4, CCL8, CCL12, and CCL17, and CXCL1, CXCL2, IL-8/CXCL8 and CXCL16” (4).

Most of inflammatory cells in solid tumors are neutrophils and their high intra-tumor density does correlate with metastasis at lymph node sites, tumor grade, and tumor stage axis (5). It has been assessed that tumor and tumor microenvironment control neutrophil recruitment and that Tumor Associated Neutrophils (TANs) can regulate tumor progression or growth control (6). Like macrophages, TANs may acquire either an antitumor activity (N1 neutrophils), either a pro-tumor activity (N2 neutrophils) (3, 7, 8). N1 activity against tumor growing and metastasis is exerted by direct or antibody-dependent cytotoxicity as well as by the activation of different innate and adaptive immune cells, including lymphocytes T and B, natural killer (NK) and dendritic cells (DCs) (9). Also, N1 TANs exhibit enhanced NADPH oxidase activity which leads to the production of reactive oxygen species, which, in turn, are cytotoxic to tumor cells (10). On the contrary, N2 TANs promote, directly or indirectly, tumor growth as well as tumor cell dissemination by secreting ECM remodeling enzymes and pro-angiogenic factors that promote metastasis and angiogenesis (11–13). Despite the considerable recent progresses in defining phenotype and functions of TANs in human cancer, the dual role of TANs in inhibiting or promoting growth and metastatic dissemination of cancer cells is still debated, arising some unresolved issues. Indeed, the most of the studies on TANs have been carried in mice models, due to the technical limitations in obtaining TANs from human tumor tissues. Unfortunately, mouse and human neutrophils are deeply different in their biology and functions. Thus, tumor growing in murine models do not accurately depict the gradual development of tumors and the complex interactions between tumor and microenvironment cells existing in humans. Moreover, while murine experimental studies highlight pro-tumor and anti-tumor functions of neutrophils in cancer, no direct evidence of pro-tumoral activity of neutrophils in human tumor tissues has been demonstrated and up to now N1/N2 polarization of TANs in humans remains uncertain (14).

In this review, we focus on the role played by TANs in pathogenesis and progression of cancer and highlight the potential role of TANs as target for novel cancer therapies.

## Neutrophil Recruitment in Tumors

**Figure 1:**
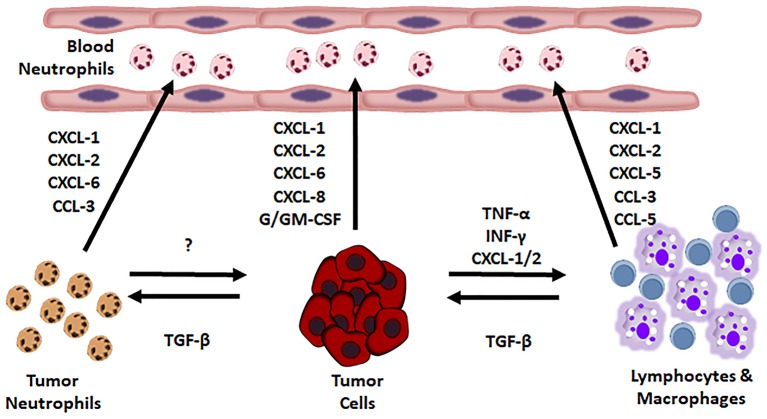
Soluble factors promoting neutrophil recruitment into the tumor.

Neutrophil recruitment is guided by some tumor produced substances (Figure 1).

By now, it has been assessed that almost every tumor type produces chemokines regulating the PMN content in microenvironment of solid tumors. Human chemokines, such as IL-8, macrophage inflammatory protein-1α (MIP-1α/CCL3) and human granulocyte chemotactic protein-2 (huGCP-2/CXCL6), murine chemokines, like MIP-1α, GCP-2 and KC (15–17), **“**behave as potent chemoattractants and neutrophil activators” (18–23). Chemokines produced by MG-63 osteosarcoma cells recruit mononuclear cells, lymphocytes and neutrophils (18). Chemokines are well regulated: R*as* upregulates CXC chemokines, causing an accumulation of neutrophils, in mice (24, 25). The cytokines “IL-1, IL-2, IL-4, IL-7, IL-10, IL-12, IFN-α, IFN-β, G-CSF, and TNF-α,” cause chemokines release *in vitro* and granulocytosis *in vivo* (26). In humans, CXCL5 (epithelial neutrophil-activating peptide-78) recruits neutrophils in Hepato-Cellular Carcinoma (HCC) promoting cancer growth and metastasis. CXCL5 appertains to a small family of secreted proangiogenic chemokine, whose expression increases in metastatic HCC cell lines and in HCC patients. CXCL5 activates the PI3K-Akt and ERK1/2 signaling pathways in HCC cells. Furthermore, it acts as a chemoattractant, *in vitro*. CXCL5 over-expression in human HCC samples does well correlate with high neutrophil content, shorter OS, and tumor recurrence (27, 28).

In tumor bearing mice, MMP-9 promotes HCC, lung and pancreatic cancer angiogenesis by promoting neutrophil recruitment (29).

In mice bearing mammary tumors, IL-17 has been demonstrated to recruit neutrophils: Interleukin (IL)-1β induces IL-17 expression from γδ T cells, which results in a G-CSF-dependent neutrophil expansion and polarization. These tumor-induced neutrophils become able to suppress cytotoxic CD8 T lymphocytes, which limit the establishment of metastases. In mouse models of spontaneous breast cancer metastasis, the neutralization of IL-17 or G-CSF and the absence of γδ T cells prevented neutrophil accumulation and down-regulated the T-cell-suppressive phenotype of neutrophils, the absence of γδ T cells or neutrophils reduces pulmonary and lymph node metastases without influencing primary tumor progression (30). Increased IL17 levels, produced by tumor-infiltrating T lymphocytes have been shown in metastatic invasive ductal breast carcinoma (IDC).

IL17 neutralization inhibits tumor cell growth and prevents neutrophil and tumor cell migration and metastasis. Pro- tumor neutrophils induce progression disease and release of CXCL1, MMP9, VEGF, and TNFα, whose reduction suppresses tumor growing. Thus, IL17 plays a pivotal role in tumor progression as emphasized by the fact that high IL17 levels do associate with shorter disease-free survival and poor prognosis in IDC patients (31).

In 238 HCC patients, the presence of neutrophils in peritumoral stroma was demonstrated. Pro-inflammatory IL-17 promotes neutrophil recruitment in peritumoral HCC tissues, via chemokines production or by activation of IL-17 producing γ δT cells (32).

Besides blood, it has been also assessed that spleen is an important reservoir of TANs. Indeed, during tumor progression, neutrophil precursors shift from spleen to the tumor stroma. On the contrary, the surgical removal of spleen delays tumor growing by reducing the number of infiltrating neutrophils, as found in a mouse model of lung adenocarcinoma presenting activation of K-RAS and inactivation of p53 (33).

Melanoma metastasis formation is critically increased by “High Mobility Group Box 1 protein (HMGB1)” released by UV irradiated keratinocytes. This effect depends on the activation of neutrophils, by the release of HMGB1 from UV-damaged epidermal keratinocytes and driven by Toll-like receptor 4 (TLR4). In a engineered mouse model, it was ascertained that UV-induced inflammatory response stimulates angiogenesis, via neutrophil activity, and promotes melanoma cells migration toward endothelial cells. Thus, inflammatory response to UV-irradiation catalyzes reciprocal melanoma-endothelial cell interactions leading to perivascular invasion, a phenomenon originally described as “angio-tropism” by histo-pathologists (34).

LTB4, a leukotriene early mediator of inflammation, produced by mast cells of silica exposed lungs, is able to recruit neutrophils by interacting with BLT1, a leukotriene B4 receptor, on neutrophils and stimulates rapid tumor growth. Deletion of BLT1, on the contrary, delays tumor growth in an implantable lung tumor model (35).

Another mechanism to modulate neutrophils infiltration of tumor microenvironment, is to modify their life span. In Head and Neck Squamous Cell Carcinoma (HNSCC), neutrophils have reduced apoptosis, due to the secretion of macrophage migration inhibitory factor (MIF) by tumor cells, as demonstrated in Trellakis et al. (36). Moreover, the up-regulation of autophagy in neutrophils may represent a mechanism by which immune cell activation is associated to tumor progression. Autophagy is associated to numerous physiological and pathological processes, including cell survival, death, and metabolism (37). Autophagy occurs also in cancer, as the result of chronic hypoxia and inflammation (38), in order to enable cancer cell survival under stress condition (39, 40).

Neutrophils infiltrating human HCC showed enhancement of autophagy through the activation of Erk1/2, p38, and NF-κB signals. This mechanism strongly increases the survival and the pro-tumor effects of neutrophils in HCC (41). IFN-β regulates apoptosis of pro-angiogenic tumor infiltrating neutrophils and influences either extrinsic either intrinsic apoptosis pathways. The life span of TANs is remarkably prolonged in tumor bearing Ifnb1(-/-) mice, compared to wild type controls. When apoptosis is inhibited, an increase of longevity and the accumulation of tumor infiltrating neutrophils occurs, in the absence of endogenous IFN-β (42).

Moreover, neutrophils migration is influenced by IFN-β via CXCL1, CXCL2, and CXCL5 chemokines and their receptors. CXCL1 and CXCL2 gradients are found in tumor-bearing mice, low level of chemokine is found in bone marrow (BM) and high level in the tumor. On the contrary, CXCR2 expression was higher on neutrophils from BM and lower in TANs. IFN-β is not really able to regulate CXCR2, but it regulates the CXCR2 ligands. Thus, IFN system can act as effector in natural cancer surveillance (43).

In a same way, using highly purified neutrophil from healthy donors, it has been demonstrated media from thyroid cancer cells were demonstrated to stimulate neutrophils chemotaxis by IL-8, and their survival by GM-CSF. Many changes in morphology and activation of neutrophils (e.g., CD11b and CD66b up-regulation and CD62L shedding) were generated. Moreover, expression of pro-inflammatory mediators like IL-8, VEGF-A, and TNF-α, and releasing of MMP-9 were induced (44).

## Neutrophil Polarization in Tumor Tissues

Neutrophils are present in the microenvironment of many solid tumors, e.g., melanoma, advanced gastric carcinoma and childhood brain tumors (45–47). A detailed study in human primary and metastatic melanoma infiltrates assessed the presence of NK, macrophages and neutrophils, representing 80% of the infiltrated cells (45).

**Figure 2:**
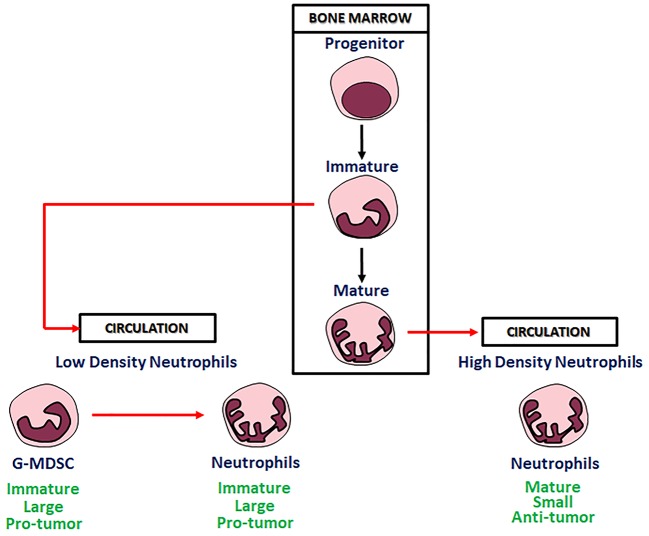
Neutrophils in bone marrow and circulation.

In microenvironment factors derived from tumor cells are able to modify phenotype and function of myeloid cells into phenotypically distinct “sub-populations.” Substantial plasticity has been evidenced in these sub-populations. Cytokine signals, epigenetic modifications and other microenvironment factors can modify morphology and functions of these cells, often substantially. These data show that the regulation of the differentiated hematopoietic cells by microenvironment factors, including those generated during immune responses, is a common mechanism for innate or adaptive immunity modulation (48, 49). Existence of neutrophils polarized to N1 TANs and N2 TANs phenotypes with anti-tumor and pro-tumor functions, has been documented in tumor bearing mice (3). The N1 neutrophils are short-living, highly cytotoxic cells and show a mature phenotype and high immune-stimulating activity. On the other hand, the N2 neutrophils, are long-living, low cytotoxic cells, showing an immature phenotype, and a high pro-angiogenic, pro-metastatic and immunosuppressive activity (Figure 2).

Thus, tumor microenvironment factors do regulate neutrophil plasticity. In particular, transforming growth factor-β (TGF-β), an immunosuppressive cytokine has been demonstrated to be one of the main modulators of neutrophil polarization, in mice. TGF-β is produced by tumor and immune cells, and can act on many components of the immune system. Due to the importance of TGF-β, many recent studies speculate that blocking the TGF-β-induced signaling in the tumor microenvironment, could enhance anti-tumor immunity and anti-cancer therapy (50).

**Figure 3:**
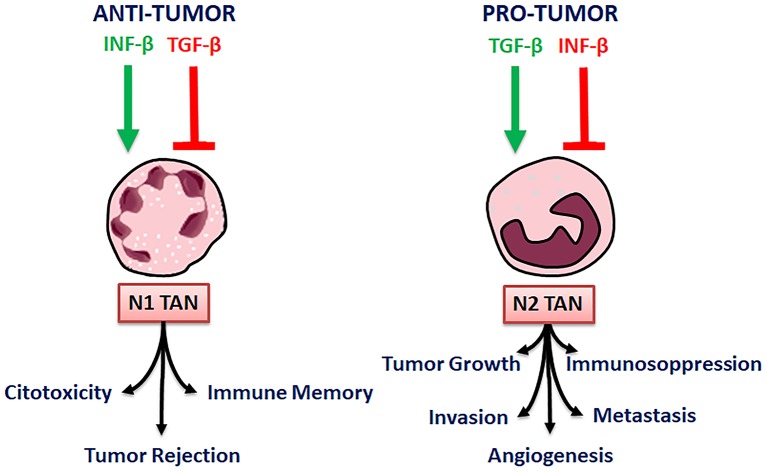
N1 and N2 TAN polarization and their activity.

When TGFβ activity is blocked, N1 neutrophils become cytotoxic toward tumor cells and activate CD8^+^ T cells, whereas N2 neutrophils re predominant in the control animals (51). Accordingly, TGF-β by itself promotes N2 pro-tumorigenic phenotype in neutrophils, in mice. Besides TGF-β, neutrophil polarization is promoted also by the cytokines IFN-β. IFN-β stimulates N1 while inhibits N2 polarization, TGF-β, on the contrary, stimulates N2 and inhibits N1 phenotypic polarization. By blocking TGF-β, a significant slowing of tumor growth is reached, through many mechanisms, comprising of CD8+ T cells and macrophages activation (Figure 3) (3).

In addition to tumor cells, tumor microenvironment cells are also able to regulate neutrophil activity in cancer and modulate cancer niche, since tumor microenvironment evolves as tumor progresses (52). So, it has been demonstrated that IL-6 produced by gastric cancer mesenchymal stem cells (GC-MSC) determines, in gastric cancer, neutrophil N2 polarization, regulating their chemotaxis, survival, activation, and function via an “IL-6–STAT3–ERK1/2 signaling cascade” (53).

After co-culture with mesenchymal stromal cells (MSCs) primed by TNF-α, neutrophils isolated from bone marrow of normal mice or spleen of tumor-bearing mice acquire immunosuppressive function *in vitro* and induce tumor progression *in vivo*. So, MSCs make neutrophil immunosuppressive and tumor-promoting (54). In mouse tumor models, it has been assessed that neutrophils show different heterogeneity and plasticity, and assume N1 or N2 phenotype and function, according to the different tumor progression time. In mouse tumor models, it has been assessed that neutrophils show different heterogeneity and plasticity, and assume N1 or N2 phenotype and function, according to the different tumor progression time. In two different models of murine tumor cell lines, Lewis lung carcinoma (LLC) and AB12 (mesothelioma), it has been demonstrated that neutrophils change their phenotype and function, according to tumor niche formation and tumor progression. In the beginning of tumor development, neutrophils are in the peritumoral tissue, are more cytotoxic and produce more NO and H_2_O_2_. Later on, neutrophils enter the tumor tissue and exert pro-tumorigenic activity (55).

Neutrophils are very heterogeneous in their phenotype and functions. Sagiv and collaborators identified in human cancer blood three different distinct populations of circulating neutrophils: “a heterogeneous subset of low-density neutrophils (LDNs) that is present transiently in self-resolving inflammation but accumulate in both tumor bearing mice and cancer patients, mature high-density neutrophils (HDNs) and immature myeloid-derived suppressor cells (MDSCs).” In tumor bearing mice, HDNs are capable of switching to a TGF-β-triggered LDNs phenotype with immunosuppressive properties, similar to those of MDSCs (56) (Figure 2). The same Authors described similar neutrophil populations in late stage tumor bearing mice in which “HDN to LDN transition occurs spontaneously” and immature myeloid cells retain immunosuppressive effects (56).

By comparing the RNA profiles of TANs and the CD11b^+^/GR1^+^ MDSC granulocytic fraction from tumor bearing mice with bone-marrow naïve neutrophils from non-tumor-bearing animals, used as baseline control, Fridlender and colleagues showed that genetic profiles of TANs, neutrophils and granulocyte precursors differ markedly. These differences result in the reduction of cytoskeleton organization, anti-bacterial functions and granule protein production and are probably due to the fact that TANs, do not need to move, once infiltrated into tumor (51). Whole-genome profiles of human PNM-MDSCs peripheral blood samples from healthy donors and tumor patients, allowed Condamine et al. to assess that PMN-MDSC have a distinct genomic profile either from PMNs isolated from the same cancer patients and PMNs from healthy donors (57).

## The Anti-Tumor Role of Neutrophils

**Figure 4:**
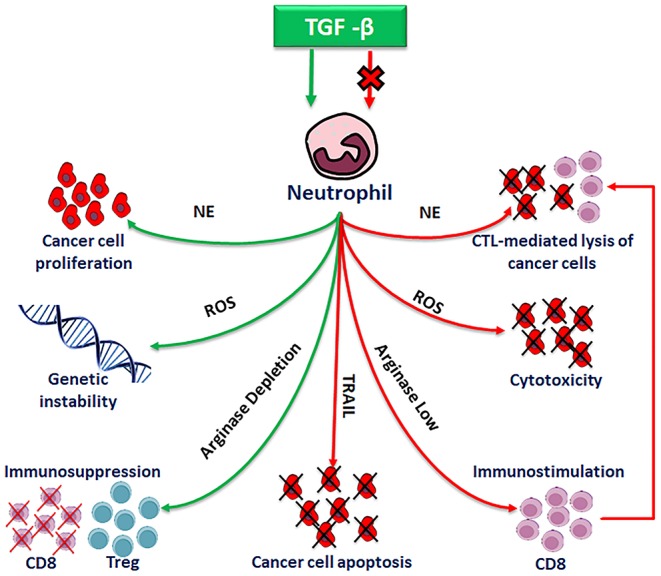
Pro-tumor and anti-tumor functions of polarized neutrophils.

TANs are involved in antitumor activity as well as in tumorigenesis, tumor progression and metastasis, either *in vitro* and *in vivo*, as depicted in Wu et al. (58) (Figure 4).

Neutrophils are cytotoxic to tumor cells both *in vitro* and *in vivo* (59, 60). It has been demonstrated that a colon adenocarcinoma cell line, transfected with G-CSF, lost tumor characteristics due to neutrophil accumulation at the tumor site. Interesting thing, neutrophils could discriminate between G-CSF-producing and G-CSF-non producing cells and selectively inhibit only the G-CSF-producing tumor cells (61).

There are evidences that Reactive oxygen species (ROS), myeloperoxidase (MPO), H_2_O_2_ and proteases produced by neutrophils as antimicrobial agents, have also a potential antitumor activity. Recent investigations on animal models and preliminary clinical studies, have highlighted the potential antitumor role of polymorphonuclear neutrophils (PMNs). PMNs from some healthy donors have been demonstrated to exert a naturally potent cytotoxic activity against four human cancer cell lines (62). The cytotoxic activity is specific for cancer cells, since neutrophils are not cytotoxic to primary normal epithelial cells or an immortalized breast epithelial cell line. The transfection of the immortalized mammary cells with plasmids expressing rat sarcoma viral oncogene homolog (Ras) and teratocarcinoma oncogene 21 (TC21) oncogenes promotes an aggressive phenotype in PMNs. Also, it has been shown that neutrophils from healthy donors reduce growing of tumor and increase mice survival, when administrated to tumor-bearing animals (63).

Moreover, the proto-oncogene c-met (MET) is necessary for neutrophil attraction. Indeed, its deletion in mouse neutrophils elicits tumor growing and metastasis and reduces neutrophil infiltration either in primary tumor and metastatic sites. It has been demonstrated that MET, induced by tumor inflammatory stimuli, promotes neutrophil migration across endothelium and production of inducible nitric oxide synthase (NOS) after c-met ligand Hepatocyte Growth Factor (HGF) stimulation. Consequently, HGF/MET-dependent release of nitric oxide (NO) by neutrophils promotes cancer cell killing, which highly reduces tumor growth and metastasis (64).

N1 TANs are also able to exert antitumor activity by eliciting antitumor immune responses. In fact, neutrophils recruit and activate immune cells (9, 65–68) by producing a variety of chemical factors such as cytokines, chemokines, and proteases, able to stimulate T cells proliferation, NK and dendritic cells maturation (69). Finally, even if neutrophils help metastasis formation by preparing the metastatic niche, N1 TANs can prevent metastases formation, by producing cytotoxic substances.

In a mouse model of metastatic renal cell carcinoma (RCC), it has been shown that neutrophils recruited to the lung built an antimetastatic barrier with loss of neutrophil chemokines in tumor cells, thus limiting lung metastatic seeding (70). Similarly, in breast cancer, H_2_O_2_ produced by neutrophils is able to inhibit metastases formation, by preventing breast tumor cells seeding in the lung Neutrophils have been demonstrated to cumulate in the lung prior to the arrival of metastatic cells in mouse models of breast cancer (10). Moreover, high infiltration of TANs has been shown in human CRC tissues, higher TAN density being associated to a better prognosis. A higher neutrophil density in stage III patients was also shown to associate with high responsiveness to 5-FU (71). Loffredo et al. showed that neutrophils from healthy donors release the antiangiogenic isoform VEGF_165b_, and suggested that VEGF_165b_ could exert an antitumor activity in lung cancer (72). Finally, in a murine sarcoma model, neutrophils have been shown to be essential for the activation of an IFNγ-dependent pathway of immune-resistance to 3-methylcholantrene-induced primary carcinogenesis, associated to polarization of a subset of CD4^−^ CD8^−^ unconventional αβ T cells. Moreover, in undifferentiated pleomorphic sarcoma, neutrophil infiltration associates with a type 1 immune response and a better clinical outcome (73).

## The Pro-Tumor Role of Neutrophils

### N2 TANs and Tumorigenesis

Recently the role of N2 polarized TANs in tumor development has been widely investigated (74). It has been ascertained that neutrophils produce and release genotoxic DNA substances, that increase DNA instability. In fact, neutrophils are release genotoxic ROS and NO in tumor microenvironment. Haqqani and coworkers demonstrated that in a mouse model of subcutaneous tumors, the number infiltrating neutrophils into tissue tumor correlates with the number of mutations at the hypoxanthine phosphoribosyltransferase (*Hprt*) locus (75). In the same syngenic mouse model, inducible NOS (iNOS) was detected mainly in neutrophils, while NOS was found in tumor homogenates. iNOS and NOS contents, neutrophil infiltration statistically correlates with frequency of mutations. To study the increase of the number of infiltrating neutrophils, tumors were \significant increase in neutrophil content was observed, correlating with frequency of mutation. Due to the fact that neutrophils are considered a source of genotoxic ROS or NOS, these results strongly suggest that tumor-infiltrating cells may be mutagenic and can contribute to the genetic abnormalities associated with tumor progression, in the mutatect murine tumor model (76).

Furthermore, it has been assessed that neutrophils recruited to chronic inflammation sites may be tumorigenic through multiple mechanisms. ROS released by neutrophils during chronic inflammation, like hypochlorous acid (HOCl), formed by MPO, cause DNA damage and are mutagenic in lung cells, *in vitro*. HOCl is one of the major neutrophilic oxidant. It has been ascertained that MPO catalyzed formation of HOCl during lung inflammation is a significant source of neutrophil-induced genotoxicity. Neutrophils can cause DNA damage through release of ROS, and gene mutation in premalignant epithelial cells, thus driving oncogenic transformation in lung cancer (77, 78). Also, at physiological concentration, HOCl induces mutations in the HPRT gene, determining three major types of DNA lesion. Depletion of circulating neutrophils highly reduced pulmonary MPO activity as well as 3-(2-deoxy-beta-D-erythro-pentofuranosyl)pyrimido[1,2-alpha]purin-10(3H)-one (M (1)dG) adducts to DNA, thus providing a causal link between neutrophils/HOCl and pulmonary genotoxicity, *in vivo* (79).

In an *in vitro* co-culture model mimicking intestinal inflammation in ulcerative colitis, it has been demonstrated that in the colon epithelial cells, neutrophils increase errors in cell replication.

In chronic colon inflammation, “activated neutrophils cause an accumulation of target cells in G2/M, consistent with the installation of a DNA-damage checkpoint” (80).

A role of neutrophils has been also assessed in hepatocarcinogenesis, in which chronic inflammation has been demonstrated to be the major etiological factor. In a transgenic zebrafish model, the role of neutrophils has been investigated by inducible expression of oncogenic k-ras (V12) in hepatocytes. Upon induction of k-ras (V12), neutrophils recruitment to the liver has been observed. Time-lapse video analysis showed that neutrophils present a slow migratory pattern along the tumor edge, whereas are relatively stationary upon entering the k-ras (V12)-expressing liver. The fluorescence-activated cell sorting of HCC cells and TANs showed a pro-inflammatory microenvironment, with increased TGF-β1a expression in k-ras (V12)-expressing hepatocytes and a loss of anti-tumor activities in TANs (81) MPO.

Neutrophils can accelerate tumorigenesis. Coussens et al. demonstrated that MMP-9, supplied by bone marrow–derived cells, contributes to skin carcinogenesis (82). On the other hand, in mammary tumors, systemic depletion of neutrophils was demonstrated to prevent tumor formation. In a Helicobacter hepaticus-infected mouse model it was shown that the systemic depletion of neutrophils, by anti-Ly-6G antibody entirely inhibited the mammary tumor development (83).

### N2 TANs and Tumor Growth

Neutrophils produce and secrete many substances able to stimulate cell proliferation, either *in vitro* and *in vivo*.

Neutrophil secreted PGE2 promotes non- small cell lung cancer (NSCL) proliferation by direct cell-to-cell contact. This interaction promotes inflammatory mediators release, promoting, in turn, cell proliferation, which can be attenuated by neutrophil elastase (NE) activity inhibition (84).

In lung, silica-induced pulmonary inflammation causes silicosis, eventually leading to lung cancer. In BLT1(-/-)K-ras(LA1) mice, crystalline-silica exposure induces either production of LTB4 by mast cells and macrophages and neutrophil recruitment (35).

In lung cancer, inflammatory tumor-associated cells influence tumor growing and invasiveness, as demonstrated in an LSL-K-ras-Elane (-/-) mice. “NE induces tumor cell proliferation by NE insulin receptor substrate-1 (IRS-1) degradation. After IRS-1 degradation, an increased interaction between PI3K and PDGFR occurs, thereby skewing the PI3K axis toward tumor cell proliferation. This relationship was also identified in human lung adenocarcinomas” (85).

In mice, lung cancer promotion has been related to inflammation, by the activation of the IL-8 pathway and recruitment of neutrophils and release of NE (86).

Colon inflammation contributes to colorectal cancer development, by modulating the expression in tumor lesions of cytokines such as TNFα and IL6 and chemokines, i.e., CXCL1, prostaglandins, COX-2, and Wnt5A (Wnt Family Member 5A). The elevation of these substance amount is suppressed in prostaglandin EP2- (PGE2-EP2) deficient mice. PGE2-EP2 stimulation in cultured neutrophils increases TNFα, IL6, CXCL1, COX-2, and other pro-inflammatory genes expression. PGE2-EP2 expression in infiltrating neutrophils has been also demonstrated in clinical specimen of colorectal cancer associated ulcerative colitis (87).

It has been also demonstrated that after an acute wound, like, for instance, a tumor biopsy, neutrophils are recruited and participate in increasing tumor mass by PGE2 production (88).

In glioblastoma, neutrophils are recruited during anti-VEGF therapy. Neutrophils promote an increased glioblastoma cell proliferation rate, upregulated by augmented S100A4 gene expression. S100A4 downregulation *in vitro* and *in vivo* blocks tumor progression. Moreover, S100A4 depletion increases the efficacy of anti-VEGF therapy in glioma (89).

Also, infiltrating neutrophils are able to induce RCC progression, by modulating androgen receptor/cMyc signals and the expression of related cytokines (90). Neutrophils promote follicular (FL) and diffuse large B cell lymphomas growth. In this malignancy, neutrophils and stromal cells cooperate to sustain FL B-cell growth, through the BAFF/APRIL pathway. Moreover, neutrophils activate stromal cells in a NF-κB-dependent manner, indirectly, promoting malignant B-cell survival (91).

Finally, neutrophil α-defensins induced an increased proliferation of A549 alveolar carcinoma cells in a MAP kinase-dependent fashion. The induced proliferation was eliminated by an anti-defensin neutralizing antibody (92).

### N2 TANs and Tumor Metastasis

Neutrophils are also involved in cancer metastasis, as they promote tumor cell migration, invasion and extracellular matrix degradation (93). In particular, they participate to the constitution of the metastatic niche (94) and to the acquisition of metastatic phenotype in certain tumor cell type. Tazawa et al. demonstrated, in a mouse model, that non-metastatic fibrosarcoma cells acquire a metastatic phenotype, when implanted subcutaneously in mice at once with sponge determining inflammation. This phenomenon is associated to neutrophils increase. Using a selective monoclonal antibody, neutrophils depletion either in blood circulation and at the local inflammation site occurred, and a significant reduction of metastasis was observed (95).

Metastasis enhancement is often promoted in an indirect way. In mice, bacterial lipopolysaccharide (LPS) recruits neutrophils which, in turn, produce proteinases able to facilitate lung metastases. Inflammation of the lung stimulates recruitment of bone marrow-derived neutrophils, releasing Ser proteases, elastase and cathepsin G. This results in the proteolytic destruction of the antitumorigenic factor thrombospondin-1 (Tsp-1). The ablation of these proteases protects Tsp-1 from proteolysis and suppresses lung metastasis (96).

In a coculture model, oncostatin M, released by GM-CSF stimulated neutrophils, has been shown to increase breast tumor cell detachment, to promote invasiveness of MDA-MB-231 and T47D human cells and to induce breast cancer progression (97).

Hyaluronan produced by tumor cells activates neutrophils which promote tumor cell motility (98). MIF promotes migration of human HNSCC cells. Neutrophil infiltration is proportional to tumoral MIF levels (99). Neutrophils promoting androgen receptor (AR)/MMP-13 signals are implicated in bladder cancer cell invasion (100). VEGFa/hypoxia-inducible factor (HIF) 2α and estrogen receptor β activation, induced by neutrophils, promote renal carcinoma metastasis (101).

Moreover, neutrophils are able to determine epithelial to mesenchymal transition, so to enhance tumor cell migration and invasion ability (102–104).

Several studies have demonstrated a direct correlation between the release of interleukin IL-8 by tumor cells *in vitro*, and tumor growth and metastatic potential in mice tumor models. It has been speculated that the cellular response to IL-8 released by tumor cells enhances angiogenesis and contributes to tumor growing and progression. The substances released by the responding neutrophils could promote tumor cell migration through the extracellular matrix, help them enter vessels and reach metastatic sites. Moreover, ROS produced by neutrophil oxidases to kill pathogens can interact with tumor cells, attenuate apoptotic cascade and increase their mutational rate (105).

In addition to metastasis promotion, neutrophils can carrier tumor cells in order to promote extravasation and enhance tumor metastasis in advanced malignancies (58, 106, 107).

In pancreatic ductal adenocarcinoma, it has been observed that circulating tumor cells (CTCs) are surrounded by white blood cells (WBC), thus hypothesizing a relationship between WBCs and CTCs in metastasis formation (108).

A similar function has been hypothesized for circulating TANs (cTANs). In 180 advanced cancer patients with various types of cancer, cTANs have been analyzed by flow cytometry. Also, a murine model mimicking advanced tumor was studied and circulating neutrophils were analyzed by gene transcriptional analysis. Either in patients and mice peripheral blood, cTANs were increased and inhibited the activation of the peripheral leukocytes. Authors propose that this abundance of cTANs contributes to the CTC survival by the suppression of peripheral leukocyte activation (109).

### N2 TANs and Tumor Angiogenesis

Neutrophils play a pivotal role in activating angiogenesis in a previously quiescent tissue during the early stages of carcinogenesis.

The role of neutrophils in tumor angiogenesis was assessed for the first time in human biopsies (110). In myxofibrosarcoma patients, numerous neutrophils were observed in high malignant tumors and a correlation does exist between a high number of neutrophil and intra-tumor micro-vessel density (111). An even stronger evidence was shown in animal models. In subcutaneously injected nude mice neutrophil infiltration in human Bowes melanoma cells, transfected to over-express CXCL6, was 10 times higher than in controls, and an increase of angiogenesis was observed (16).

Now, the function of neutrophils in tumor angiogenesis has been deeply ascertained (112). Neutrophils are able to release molecules that activate endothelial cells and promote angiogenesis (110, 113), including Bv8, expressed in the bone marrow, which promotes either angiogenesis and haematopoietic cell mobilization. In a mouse model, the implantation of CD11b+Gr1+ tumor myeloid cells, results in Bv8 up-regulation “Anti-Bv8 antibodies” reduce “mobilization of CD11b+Gr1+ cells stimulated by G-CSF,” inhibit growing of several tumors in mice and suppress tumor angiogenesis (114).

Neutrophils are plenty of MMP-9 and lack the endogenous tissue inhibitor of metalloproteinases (TIMP). Using MMP-9, neutrophils promote angiogenesis. Pro-MMP is even more potent. In order to induce angiogenesis by neutrophil proMMP-9, activation of TIMP-free zymogen and the catalytic activity of the enzyme are needed. This is confirmed by the fact that the generation and purification of stoichiometric complexes of neutrophil proMMP-9 and TIMP-1 failed to induce angiogenesis. So, neutrophil-derived MMP-9 can exert pro-tumor activity (115).

MMP-9 is functionally involved in VEGF activation, chronic angiogenesis induction, tumor growth at early stage. During pancreatic carcinogenesis, neutrophils which express MMP-9 do infiltrate angiogenic islet dysplasia and tumors, whereas macrophages expressing MMP-9- are situated at the edge of the lesion. Depletion of neutrophils significantly suppresses the association between VEGF and VEGF-receptor (29, 115, 116). Moreover, it has been shown that primary tumors like high aggressive human fibrosarcoma and prostate carcinoma recruit infiltrating MMP-9-positive neutrophils, exhibiting enhanced angiogenesis and intravasation. Specific neutralization of neutrophils by IL-8, implies the reduction of angiogenesis and intravasation, both of which are rescued by purified neutrophil proMMP-9. It has also been demonstrated that neutrophils TIMP-free MMP-9 regulate tumor angiogenesis and intravasation (117).

### N2 TANs and Tumor Immunosuppression

Immune response contributes to orchestrating tumor progression. In tumor microenvironment numerous immune cells actively interact with the tumor cells. At the beginning, innate and adaptive immune cells cooperate to eradicate micro-colonies of tumor cells (118). In advanced tumor mouse models, TANs induce CD8 T-cell apoptosis via TNFα pathway and NO, and so do participate to the immunosuppressive response. TANs and granulocytic myeloid-derived suppressor cells (G-MDSCs) suppress CD8 T-cell proliferation, influence their activation and abolish the anti-tumor effect of CD8 T cells (119). Neutrophils, too, modulate this process. TANs are able to inhibit T cells proliferation by releasing arginase 1 (ARG1) and modulating PD-L1/PD-1 signaling, in order to regulate an immunosuppressive response. MDSCs producing arginase 1 are higher than normal in RCC and NSCLC peripheral blood patients. Therefore, these cells do play an important role in tumor escape mechanism (120, 121).

PMN-MDSCs are key regulator cells of the immune response in cancer, are produced in a great number in tumors and are directly involved in promoting tumor progression. However, their heterogeneity and the lack of specific markers frustrate the complete comprehension of their biology and clinical significance. This population of immune suppressive cells is identified in mice by the expression of the surface markers CD11b and GR1 and the ability to inhibit T lymphocyte activation. Peranzoni and co-workers identified in tumor mice the immune suppressive “CD11b^+^/GR1^+^ MDSC” population that includes at least two different subsets: the “granulocytic (Ly6G^+^)” and “monocytic cells (Ly6C^+^),” possibly with different immunosuppressive properties (122).

The interaction between neutrophils, TANs and PNM-MDSCs during tumor progression is unclear, though PMN-MDSCs have in common with N2 neutrophils some markers and show similar immunosuppressive activities (14). Condamine et al. recognized “the Lectin-type oxidized LDL receptor 1 (LOX1) as a marker for distinguishing PNM-MDSCs” from neutrophils in blood samples from healthy donors and tumor patients. They found that “low density PMN-MDSCs and HDNs from the same cancer patients had a distinct gene profile” and that unlike LOX-1^−^, LOX-1^+^ neutrophils in tumor tissues show an effective “immune suppressive activity, up-regulation of ER stress, and other biochemical characteristics of PMN-MDSCs” (57). Anyway, much studies have to be performed on this specific issue.

In tumors, hypoxia promotes a strong and selective pressure leading to angiogenesis, metastatic niche formation, invasion, and metastatic spread of tumor cells (123).

In primary lung cancer, hypoxia promotes the release of cytokines and growth factors which can create a pre-metastatic niche through the recruitment of CD11b+/Ly6Cmed/Ly6G+ myeloid cells. Also, hypoxia reduces the cytotoxic functions of NK cells, as shown in mice injected “with cell-free conditioned medium, derived from hypoxic mammary tumor cells” (124). Moreover, it has been now well assessed that immune cells promote the initial metastatic dissemination of carcinoma cells. Neutrophils promote tumor cell survival and extravasation at sites of metastatic dissemination, and, in particular, CD11b(+)/Ly6G(+) neutrophils enhance metastasis by inhibiting functions of NK cells, so significantly increasing the intraluminal tumor cell survival. Thereafter, neutrophils facilitate the extravasation of tumor cells, by the secretion of IL1β and matrix metalloproteinases (125). In a breast cancer mouse model, it has been demonstrated that invasive breast cancer cells are able to reprogram myeloid cell differentiation in the bone marrow, so generating immunosuppressive neutrophils by the stimulation of a tumor-derived G-CSF (126).

A great number of neutrophils, expressing high levels of immunosuppressive PD-L1, was found in 105 Gastric Cancer (GC) patients. PD-L1^+^ expression was activated by tumor-derived GM-CSF, “via Janus kinase (JAK) -signal, by the activation of a signal transducer and activator of transcription 3 (STAT3) factor pathway.” The PD-L1^+^ neutrophils suppressed normal immunity of T-cell *in vitro* and promoted. the progression the progression of the disease, reducing GC patient survival. By blocking PD-L1 on these neutrophils, this effect could be reversed (127). Finally, PD-L1 expression on neutrophils does contribute to the impaired immune response in infectious disease. It has been also ascertained that neutrophils do negatively modulate adaptive immunity via PD-L1/PD1 signaling, in human HCC and hepatoma bearing mice (128).

## Neutrophils and Tumor Prognosis

A large body of evidence shows that neutrophil number in blood stream and tumor tissues of cancer patients does correlate with prognosis of the disease. Moreover, great neutrophil density in tumors is regarded as an independent index of poor prognosis (5). In many tumors, in fact, it has been assessed such an association: a poor outcome has been observed in RCC and a negative prognosis index of intra-tumor neutrophils has been assessed in metastatic RCC. In localized clear cell RCC (129), the presence of intra-tumor neutrophils strongly indicates short recurrence-free survival (RFS), cancer-specific and overall survival, and can be regarded as an independent prognostic factor. In 121 patients subjected to nephrectomy for localized RCC, intra-tumor CD66b+ neutrophils, CD8+, CD57+ immune cells, and carbonic anhydrase IX [CA IX]) were assessed by immunohistochemistry. The intra-tumor neutrophils ranged from zero to 289 cells/mm^2^ tumor tissue. The presence of intra-tumor neutrophils was associated to tumor size increasing, low hemoglobin, high creatinine, and CA IX < or = 85%. In a multivariate analysis, the presence of intra-tumor neutrophils and low hemoglobin were prognosis indexes significantly associated with short RFS (129).

The overexpression of CXCL5 by itself or plus the presence of intra-tumor neutrophils, is an independent prognostic indicator of overall survival and cumulative recurrence, as suggested by multivariate studies (27, 28).

CXCL5 promotes neutrophil infiltration and poor prognosis in HCC. In HCC patients and in hepatoma-bearing mice, neutrophils have been shown to infiltrate the peritumor tissue. The neutrophil-to-lymphocyte ratio (NLR) was higher in peritumor than in intratumor tissue. This negatively correlates with the OS of HCC patients. A high percentage of the infiltrating neutrophils is positive for PD-L1. In peritumor tissue the ratio of PD-L1^+^neutrophils-to-PD-1^+^T cells was higher and associated to the DFS. Indeed, the PD-L1^+^ neutrophils from HCC patients, were able to suppress T cell proliferation and activation, being these actions partially reversed by the blockade of PD-L1. Thus, tumor microenvironment modulates antitumor immunity PD-L1 expression, by infiltrating neutrophils (128).

The HGF, a pleiotropic cytokine produced by neutrophils infiltrating bronchioloalveolar carcinoma (BAC) and activating the proto-oncogene c-met, is an index of poor prognosis in various tumors, including BAC. HGF promotes tumor BAC cell spreading and worsens its prognosis. GM-CSF and TGF-α present in the lung tumor microenvironment, promote the release of HGF from intratumor neutrophils. Immunoreactive HGF has been found in bronchoalveolar lavage fluid (BALF) supernatants from 34 out of 36 patients, whereas it was not detectable in BALF from healthy controls. “In immunocytochemical studies of BALF cytospin preparations and tumor specimens from the patients, neutrophils were always HGF-positive” (130). In a retrospective study on HNSCC, Trellakis and coworkers demonstrated that tumor tissue is considerably infiltrated by PMN granulocytes, and that a high infiltration of these cells do associate with low survival in advanced disease. PMN granulocyte count, NLR and the levels in serum of IL-8, CCL4 (MIP-1β) and CCL5 (RANTES) have been found significantly higher in the peripheral blood of HNSCC patients than in that of controls (131). In primary melanomas from 186 stage-I/II surgically resected melanoma patients, infiltrating neutrophils as well as dendritic cells and T-lymphocytes were studied by immunohistochemistry. Infiltration by neutrophil and dendritic cells was independently associated with poor prognosis (132). In lung carcinoma, it has been shown that great number of preoperative circulating neutrophils, CD44(+) lymphocytes and WBCs indicate low cumulative survival. Moreover, high levels of CD44(+) lymphocytes and neutrophils do correlate with distant metastasis and prognosis in stage III/IV NSCLC patients, respectively (133).

In Colon-Rectal Cancer (CRC), increased intra-tumor neutrophils have been demonstrated to be very important for getting a malignant phenotype. The prognostic meaning of intra-tumor CD66b+ neutrophil in CRC was investigated by tissue microarray and immunohistochemistry. Numerous intratumoral CD66b+ neutrophils were found in 104/229 (45.4%) CRCs and in 29/229 (12.7%) adjacent mucosal tissues. A further analysis demonstrated that the presence of neutrophils inside the tumor does correlate with pT status, pM status and clinical stage. An univariate survival analysis, showed a significant association between great number of intra-tumoral neutrophils and shortened patients' survival was ascertained (134). Also, the presence of CD15+ neutrophils is considered an independent and unfavorable prognostic factor, in gastric carcinoma. The density of intra-tumoral CD15+ neutrophils, assessed by immunohistochemistry, associates with lymph node metastasis, metastasis and UICC (International Union Against Cancer) staging. Multivariate Cox's analysis demonstrated that the density of CD15+ neutrophils is an independent prognostic factor for OS. Kaplan-Meier analysis showed that patients with a lower number of neutrophils had more favorable prognosis than patients with more numerous TANs (135). Intra-tumor TAN density in CRC was demonstrated to have a prognostic value in Stage III 5-Fluoro Uracile treated patients. In fact, DFS in these treated patients was longer compared to DFS in untreated ones (71).

## Neutrophil to Lymphocyte Ratio (NLR) and Prognostic Evaluation

Nowadays, NLR evaluation is regarded as a very reliable prognostic index in cancer patients and many studies have been accomplished on its clinical relevance in tumors. Moreover, NLR does associate with an adverse OS in many solid tumors, so its addition to other well consolidated prognostic scores can be important for clinical decisions (136). Recently, a retrospective study in patients with epithelial ovarian cancer demonstrated the prognostic role and clinical utility of pretreatment neutrophilia and NLR. Either pretreatment neutrophilia and high NLR were independent poor prognostic indexes in these patients, being NLR more accurate than neutrophil count in predicting patients survival (137). NLR was examined also in 55 patients with advanced metastatic cancers, enlisted in a phase 1 trial and administered with anti-PD-1/PD-L1 agents. NLR was evaluated at the beginning and after the second cycle of treatment. NLR changes were demonstrated to correlate with PSF in these patients (138). Similarly, the importance as prognostic factor of NLR was evaluated in NSCLC patients treated with nivolumab. The evaluation of NLR at 2 and 4 weeks after treatment does correlate with response to treatment or PD in advanced NSCLC patients treated with nivolumab (139). Recently, NLR has been identified as prognostic factor for progression free survival in Hodgkin Lymphoma patients independently from tumor stage at diagnosis (140). In melanoma patients, it has been shown that NLR is strongly and independently associated with ipilimumab treated patients outcome (141). Finally, NLR has been demonstrated to be the only significant parameter in predicting post-surgical early complications in 188 patients subjected to liver resection for colorectal metastasis, and, so far, to improve their clinical outcome (142).

## Neutrophils and Cancer Therapeutic Strategies

**Figure 5:**
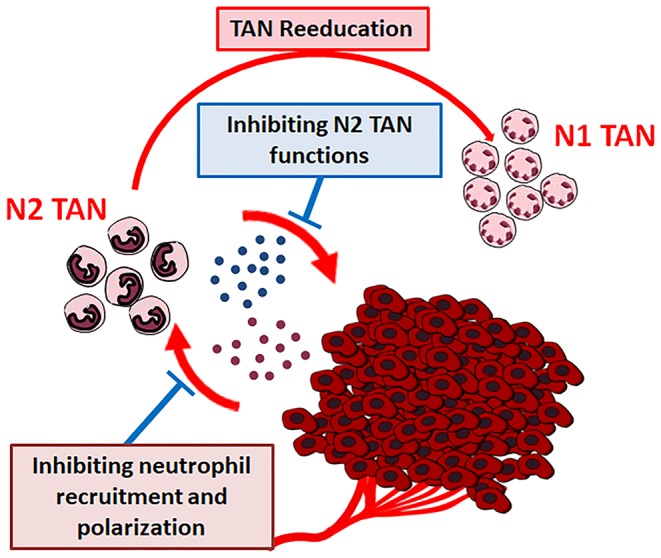
Therapeutic strategies for cancer neutrophils targeting.

The close relationship assessed between tumor microenvironment cells and cancer growth and progression, points out the possibility of novel therapeutic strategies, i.e., targeting the non-cancer-cell component of tumors (143). TANs, as well, could represent a good target for cancer innovative therapies and their use as targets for cancer therapy has been suggested (Figure 5) (13).

New approaches for cancer treatment could be the pharmacologically blocking of tumor derived factors that recruit and polarize neutrophils and/or the selective interference with the pro-tumor functions of neutrophils. These strategies could be combined with conventional or new anticancer drugs, to exert more active therapeutic effects (12).

Alyssa D. Gregory and A. Mc Garry Houghton suggest two different possible strategies: (a) targeting the “CXCL-8/CXCR-1/CXCR-2 axis,” thereby entirely exhausting TANs or (b) targeting specific PMN-derived substances able to induce tumor growing (13).

Also, “reeducation” of TANs has been proposed, by modulating the tumor microenvironment substances affecting neutrophil plasticity. As previously discussed, TGF-β modulates neutrophil N2 polarization. Condamine et al. have demonstrated that the deletion of TGF-β in myeloid cells inhibits tumor metastasis. On the contrary, the inhibited metastasis phenotype can be restored by entering back TGF-β producing myeloid cells in tumor-bearing mice. The effect is mediated by the reduction of type II cytokines, TGF-β1, arginase 1, iNOS, which promote IFN-γ expression plus the stimulation of the systemic immunity (144). It has been also demonstrated that priming of protumor neutrophils with IFN-γ and TNF-α converts neutrophil function from tumor-promoting to tumor-suppressing state, through NK cells activation, thus suggesting that the administration of normal NK cells could represent a potential therapeutic approach in tumor therapy (145).

### Preclinical Studies

Recently, have been developed cell mediated drug delivering systems. For instance, neutrophils carrying paclitaxel (PTX) containing liposomes, able to pass through the blood-brain barrier and suppress the recurrence of glioma in mice, have been engineered. In this system, inflammation factors released after tumor resection, guide neutrophil movements and trigger the release of liposomal PTX from the neutrophils, delivering it into the remaining invading tumor cells. PTX has been demonstrated to slow tumor growth recurrence, but not completely inhibit tumor regrowth (146).

NE is the major effector of neutrophil antimicrobial function (147), works on a broad variety of substrates, including cytokines, cytokine receptors, integrins, and components of the Extracellular Matrix, and promotes tumor growth, as already discussed (85). For this reason, a therapeutic strategy has been developed, using the synthetic Neutrophil Elastase inhibitor ONO-5046. In tumor bearing mice, ONO-5046 reduces tumor growth by 3-fold (85). Also, ONO-5046 inhibits, *in vitro*, the proliferation of pancreatic carcinoma cells and the progression of the tumor, whereas, *in vivo*, in a mouse model reduces human lung cancer development (148, 149). In CRC, NE has been demonstrated to be a biomarker either in patient sera or in tumor tissues, and its value has been found higher in CRC patients than in healthy people. A potential therapeutic strategy using Sivelestat, a NE inhibitor, was proposed in CRC xenografts. Sivelestat was able to inhibit tumor growth in this CRC model, suggesting that NE can be considered a putative diagnostic biomarker and a potential therapeutic target of CRC (150). NE was also studied in esophageal cancer. In patients subjected to esophagectomy, a rapid growing of occult cancer cells or tumor recurrence can occur after surgery. NE induces cell growing and invasion of five esophageal cell lines “(TE-1,−7,−8,−12 and−13).” NE stimulated “pro-transforming growth factor-alpha (pro-TGF-alpha), platelet-derived growth factor-AA (PDGF-AA), PDGF-BB” and VEGF release, phosphorylation of EGFR and triggered the ERK signaling pathway. All these factors affect cell growth. Sivelestat, significantly inhibited the NE-induced signal. transduction pathway, cell proliferation and cell invasion. Furthermore, in the medium in TE-13 esophageal carcinoma cells “Sivelestat significantly inhibited NE-induced release of TGF-alpha, PDGF-AA, PDGF-BB and VEGF. The authors postulated that postsurgical administration of Sivelestat could be used as” a new molecule in -target cancer therapy” (151).

In a similar way, NE promotes gastric cancer cell proliferation by EGFR phosphorylation, triggering the ERK1/2-mitogenic signaling pathway. Sivelestat inhibits cell growth by reducing significantly NE-induced EGFR phosphorylation and ERK1/2 activation (152). Finally, in a mammary mouse tumor model, it has been assessed that by inhibiting genetically or pharmacologically the leukotriene-generating enzyme Arachidonate 5-lipoxygenase (Alox5) the neutrophil pro-metastatic activity and lung metastasis could be reduced (153).

### Clinical Studies

Some clinical studies have been conducted trying to target microenvironment cells and TANs in tumor patients. Interferons can be used as potent clinical anti-tumor agents. Now, it is well established that type I IFNs modify neutrophil phenotype into 1 antitumor TAN 1, either in mouse models and humans. In the absence of IFN-β, pro-tumor properties dominate in neutrophils in primary lesion and pre-metastatic lung. In melanoma patients undergoing type I IFN therapy, neutrophil activation changes have been shown. Andzinski and coworkers recently found that adjuvant type I IFN therapy influences ICAM1 expression levels, without modifying the cytotoxic capacity of neutrophils. In fact, in these patients, increase of neutrophil ICAM1 expression was observed, neutrophil anti-tumor ability was potentiated, spontaneous apoptosis of isolated neutrophils was increased and immature neutrophils were uncovered in patient blood compared to controls (154).

New clinical strategies aimed to inhibit neutrophil recruitment through the inhibition of CXC receptors type 1 and 2 that are associated with the recruitment of neutrophils into tumors, have been explored (155). The phase I clinical trial (Study NCT02001974) on Triple-Negative metastatic breast cancer patients, successfully evaluated drug safety and pharmacokinetics of orally administered Reparixin, an orally available inhibitor of CXCR1 and CXCR2 in combination with paclitaxel (156). The current phase II study (Study NCT02370238) does evaluate the Progression Free Survival of newly diagnosed TNBC patients (newly diagnosed metastatic or relapsed following (neo)adjuvant chemotherapy) treated with Reparixin in combination with paclitaxel vs. paclitaxel alone, is ongoing and final results have not yet been published.

## Concluding Remarks and Discussion

The role in pathogenesis of cancer of tumor associated neutrophils (TANs) has been partly elucidated, and has become an area of intense research. After recruitment from blood stream, they are polarized in anti-tumor (N1) or pro- tumor (N2) TANs, according with tumor or microenvironment cell stimulating substances.

The evaluation of TANs as prognosis index in many tumors has been clearly assessed: high TANs number and/or elevated Neutrophil-to-Lymphocyte Ratio (NLR) do correlate a poor outcome of the patient. Due to TANs critical role in progression and metastasis, therapeutic targeting of TANs has been suggested, and many preclinical studies have been accomplished. On the contrary, a few clinical trials have been carried up to now, due to difficulty in suppressing their action for the role they have in the defense against infections and the risk of immunosuppression. Therefore, it will be necessary in the future to focus scientific efforts on these very important items.

Many of the studies presented in this review have been carried in mice, arising doubts about the validity in humans of the results assessed in mice models. Indeed, human tumors evolve very slowly and the heterogeneous tumor cell population is subjected to a broad range of selective pressures, including to escape the immunological control. Conversely, in tumor bearing mice, the xenografted cells are more genetically homogeneous and less subjected to selective pressure. Furthermore, most of the experimental studied on neutrophil functions in tumor patients, are performed on human circulating neutrophils, assuming that they are similar to the intra-tumoral ones. So, it is to be hoped that future studies will address these critical points.

## Author Contributions

MTM and MC: conception of the work and critical revision of the work. MTM and MM: extensive literature search and manuscript drafting. MC: final version approval.

### Conflict of Interest

The authors declare that the research was conducted in the absence of any commercial or financial relationships that could be construed as a potential conflict of interest.
